# Material Parameter Identification for Acoustic Simulation of Additively Manufactured Structures

**DOI:** 10.3390/ma14010168

**Published:** 2020-12-31

**Authors:** Sebastian Rothe, Christopher Blech, Hagen Watschke, Thomas Vietor, Sabine C. Langer

**Affiliations:** 1 Institute for Acoustics, Technische Universität Braunschweig, Langer Kamp 19, 38106 Braunschweig, Germany; c.blech@tu-braunschweig.de (C.B.); s.langer@tu-braunschweig.de (S.C.L.); 2 Institute for Engineering Design, Technische Universität Braunschweig, Hermann-Blenk-Straße 42, 38108 Braunschweig, Germany; h.watschke@tu-braunschweig.de (H.W.); t.vietor@tu-braunschweig.de (T.V.)

**Keywords:** acoustic black holes, acoustic-oriented design, additive manufacturing, finite element method, vibroacoustics, material parameter identification, model order reduction

## Abstract

One possibility in order to manufacture products with very few restrictions in design freedom is additive manufacturing. For advanced acoustic design measures like Acoustic Black Holes (ABH), the layer-wise material deposition allows the continuous alignment of the mechanical impedance by different filling patterns and degrees of filling. In order to explore the full design potential, mechanical models are indispensable. In dependency on process parameters, the resulting homogenized material parameters vary. In previous investigations, especially for ABH structures, a dependency of the material parameters on the structure’s thickness can be observed. In this contribution, beams of different thicknesses are investigated experimentally and numerically in order to identify the material parameters in dependency on the frequency and the thickness. The focused material is polyactic acid (PLA). A parameter fitting is conducted by use of a 3D finite element model and it’s reduced version in a Krylov subspace. The results yield homogenized material parameters for the PLA stack as a function of frequency and thickness. An increasing Young’s modulus with increasing frequency and increasing thickness is observed. This observed effect has considerable influence and has not been considered so far. With the received parameters, more reliable results can be obtained.

## 1. Introduction

The development of new measures to reduce noise in our environment is an important contribution to comfort and from a health point of view. New advanced design measures are needed to achieve these objectives. This is accompanies the challenge of using unconventional manufacturing processes and providing mechanical models for reliable simulations. This paper aims to contribute to this by new insights on material parameters.

The possibilities of integrating acoustic measures and other properties are often limited due to the given manufacturing constraints of conventional processes. With the help of additive manufacturing (AM), completely new possibilities open up. In this contribution, the additive manufacturing process material extrusion (MEX) is focused upon. If AM is used to produce structures with integrated acoustic measures or functions, design restrictions are significantly reduced. Due to the layer-wise build-up of the parts, AM allows almost every design, even a fully integrated measure. Furthermore, multiple materials can be applied directly during the manufacturing process, e.g., as additional damping material. However, mechanical models or assumptions based on experience with standard materials such as steel and aluminium are no longer sufficient. The step-wise build-up leads to anisotropies and inhomogeneities, which have to be considered depending on the individual task.

In literature, contributions exists in which the use of AM for generating structures that influence airborne and structure-borne sound is described. For example, 3D-printed tailored absorbers. Setaki et al. [1] design absorber duct lengths in such a way that the sound travelling through them receives a phase shift of 180° due to the different lengths and interferes destructively. Ring and Langer [2] link the geometric parameters of lattice structures with the resulting BIOT parameters. This allows the microstructure to be designed to achieve a specific absorber behavior. For the measures influencing structure-borne sound, new possibilities arise, e.g., for the method of Acoustic Black Holes (ABH). Here the production process is one of the biggest challenges [3]. The required thickness reduction of the structure is usually achieved with the help of milling cutters afterwards, which generates a high effort and high costs [3]. In addition, there are many restrictions regarding the placement of the ABHs and their design. Since this method reduces weight and at the same time improves the acoustic properties, it may be possible to overcome the major conflict of objectives between lightweight design and acoustics. The ABH are therefore the focus of this study as an exemplary application.

The ABH effect, first described in 1988 by Mironov [4], can be utilized in thin structures bearing structure-borne sound to focus the radiation critical bending waves in an area where they can be damped very efficiently. For this purpose, the thickness of the plate-like structures must be reduced according to a polynomial shape function. As a result of the smooth adaptation of the mechanical impedance, the amplitudes of the bending waves increase while the propagation speed decreases—an optimal area for an application of passive damping measures is formed. In 2000, Krylov published a combination of shaped area with a local damping measure and named it an Acoustic Black Hole [5].

A review of the literature since then reveals that most of the studies deal with homogeneous metal structures. Many investigations were carried out experimentally on generic plate structures [6,7,8]. There are also few studies on industrial examples, e.g., turbine blades [9] and an engine cover [10]. In addition to measurements, several numerical investigations were carried out [11,12,13,14]. However, composites and sandwich structures were also investigated with their higher design freedom. Bowyer et al. inserted an ABH exclusively into the glass fiber cover layers, in 2012 [15]. In 2017, Dorn et al. extended this approach and integrated an ABH into the sandwich core [16]. Blech et. al. showed how this can be used in an aircraft structure [17]. Further studies on carbon and glass fiber composites can be found in [18,19]. In their papers, Zhao and Prasad [20] as well as Pelat et al. [21] provide a very good overview of the current state of ABHs and their applications.

**Figure 1:**
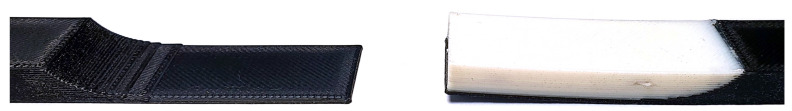
Tip of Acoustic Black Holes (ABH) beam with (**right**) and without (**left**) additional damping material.

The application of AM technologies with the highest design freedom compared to the above mentioned manufacturing techniques for design and manufacturing ABHs has successfully been demonstrated by Rothe et al. [22,23]. Even complex tube structures which act as ABHs for fluids can be produced [24]. In Figure 1 an example of an additive manufactured ABH beam with and without additional damping material is shown. The effect of the additional damping material can even be enhanced if it is designed as a constraint layer damping [25]. Such constraining layers can also be designed very efficiently by AM, e.g., by fully integrated ABHs.

Chong et al. [26] give an overview of the possibilities of how ABHs can be integrated into additive manufactured structures. They additionally show experiments and simulations on additively manufactured beams, but always assuming a constant modulus of elasticity over the frequency range. In [23], a need for frequency and thickness-dependent material parameters arise. Especially when modeling ABHs, regions of different thicknessess of the base material are obtained (as shown in Figure 1 on the left). Studies in [27] assume that a varying Young’s modulus and loss factor results depending on the thickness of the sample on which the homogenization is performed. A suitable and efficient procedure is required to identify these homogenized linear elastic parameters, which are difficult to determine due to anisotropy and strong dependencies on geometry and process parameters [27]. For this purpose, a methodological procedure is proposed in this article and demonstrated on additively manufactured beam structures. To the authors’ knowledge, the determination and consideration of the dependencies of the material parameters on the thickness have not been considered so far and offer a new approach for more reliable vibroacoustic simulations of additively manufactured structures.

In order to investigate the influence of the printed thickness, beams of different thicknesses are manufactured. Subsequently, these are characterized vibroacoustically by laser-scanning vibrometry and compared with numerical results. For efficiency reasons, the numerical models are transformed into reduced models and used for parameter study. The aim is to determine the homogenized parameters as a function of frequency and thickness.

## 2. Additive Manufacturing

AM provides a vast potential for the realization of graded properties, for instance, regarding bending stiffness and, thus, for the incorporation of passive damping measures such as ABHs due to the provision of enhanced design freedom compared to other manufacturing technologies, e.g., milling or casting. This freedom in design allows the manufacturing of complex shapes or a combination of multiple materials in order to achieve the required mechanical properties. One of the most commonly used AM technologies offering processing multiple materials in one part, without an additional joining process is needed, is MEX also referred to as fused deposition modeling [28,29]. Besides prototyping, MEX is also established in manufacturing of functional parts and end-use products [30]. This AM technology uses thermoplastic polymers as feedstock material. The material is plasticized and directed in an extrusion unit in order to build up the part’s geometry in lines or layers. A great variety of thermoplastic polymers and also thermoplastic elastomers and fiber-reinforced materials are available [28]. Because of its ease of processing and good mechanical properties, especially the resulting stiffness, polylactic acid (PLA) is one of the most frequently used materials for material extrusion and, thus, focused on in this contribution.

With AM, the creation of complex shapes is not limited to external geometries in order to achieve locally variable bending stiffness. The internal structure of a part can also be influenced, for instance, by using lattice structures with variable wall thickness, different raster angle orientations or integrated damping structures by using a combination of a stiff and a flexible material [28,31,32]. As the mechanical properties of additively manufactured parts arise during the manufacturing process, they are significantly determined by the geometry and the selected process parameters in comparison to conventional manufacturing processes [29]. In addition to process parameters, the anisotropy in mechanical properties of additively manufactured parts is also influenced by machine-specific factors such as the heated build platform or the leveling (distance between the nozzle and the build platform) [27,33]. On the one hand, this process- and machine-related anisotropy enhances the design freedom for a local adjustment of the mechanical properties. On the other hand, the modeling of additively manufactured structures is more challenging regarding the identification and quantification of the influencing factors that have to be considered.

**Figure 2:**
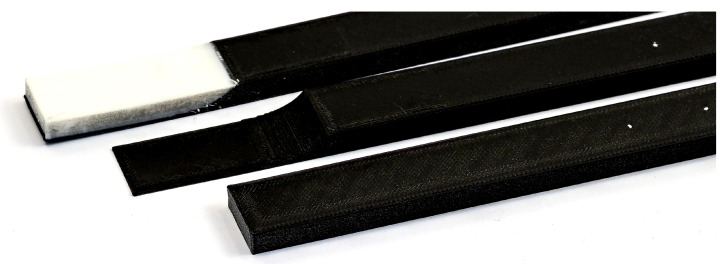
Overview of beam specimens with different system complexity: simple beam structure made of PLA (**front**), beam structure with an ABH made of PLA (**center**), and beam structure with an ABH made of PLA with an additional damping treatment made of an thermoplastic polyurethane (**back**).

In order to increase the complexity of the considered ABH systems step by step, simple beam structures made of one material without ABH and additional damping material are manufactured first (represented by the specimen at the front in Figure 2). Afterwards, they are vibroacoustically characterized and their homogenized material parameters are determined.

In Rothe et al. [23,27] it is shown that even at this step simple homogenization models are no longer sufficient. In addition to the frequency dependence of the material parameters, a dependency on the thickness respectively the number of layers can be observed.

In Figure 2 are also shown further exemplary complexity steps for the application of ABH. In the center the consideration of an ABH shape to weaken the cross-section is presented. The next complexity step (back) would be the consideration of a second material to locally increase the damping (here in white: NinjaFlex^®^ (thermoplastic polyurethane, TPU) from NinjaTek).

A valid dynamic simulation of such additively manufactured structures is only possible with material descriptions that consider both frequency and thickness dependence. This is the focus of the investigations in this paper, where a procedure for the identification of material parameters is presented. It is the necessary step towards the next level of complexity.

The chosen material for manufacturing the test specimens used for parameter identification are PLA from DAS FILAMENT (Emskirchen, Germany). The used process parameter set is shown in Table 1. The flow rate was set to 105% in order to increase stiffness due to a minimized internal void fraction. The other parameters have been selected according to the recommendations of the material manufacturer. For the manufacturing, a X400 by German RepRap GmbH (Feldkirchen, Germany) with a dual extruder system and a nozzle diameter of 0.4 mm is used. All specimens were manufactured at the same ambient temperature (23 ± 1 °C) and relative humidity (45–50%) and the feedstock materials are dried before processing in order to ensure similar manufacturing conditions.

## 3. Experimental Investigation

The experimental investigations on beam structures provide the necessary data basis for the subsequent numerical studies. In the following, it is explained which samples are to be studied and which experimental setup is to be chosen. Finally, the results are discussed.

### 3.1. Setup and Specimens

**Figure 3:**
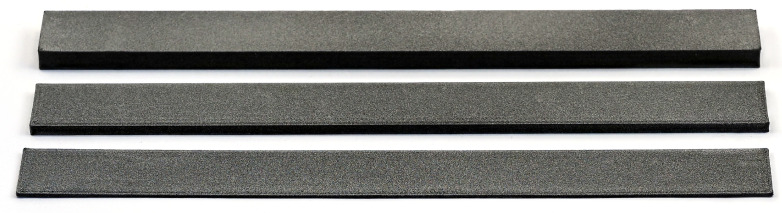
Overview of investigated specimen of three different thicknesses (6 mm, 3 mm, 1 mm).

Three different types of beam samples of the same length are produced under the conditions described in Section 2 with three different thicknesses but a constant thickness over the length. These are used for fitting the material parameters and for studying the influence of thickness. The samples are exemplary shown in Figure 3.

Each beam type is manufactured twice to get an idea of the repetitive uncertainties in vibroacoustic behavior that may arise from manufacturing imperfections. This results in six samples with the properties summarized in Table 2.

**Figure 4:**
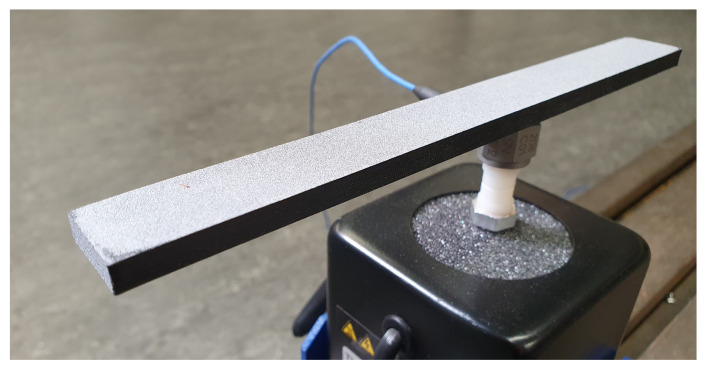
Experimental setup with 6 mm beam mounted on electrodynamic shaker.

In order to minimize external influences on the vibration behavior of the beams, they are characterized contactless by means of a laser scanning vibrometer. For this purpose, the samples are mounted eccentrically in the longitudinal direction (off-set of 0.02 m from longitudinal center) on an electrodynamic shaker in order to excite as many bending modes as possible in the considered frequency range. The surface velocities are measured. Simultaneously, the excitation force is recorded with a force sensor mounted directly at the force transmission point between shaker and beam. The setup is illustrated in Figure 4.

Due to the dark and highly light-absorbing PLA, the surfaces of the beams are sprayed with a reflection spray so that measurement with the laser vibrometer is possible. This can be seen as a grey top coat on the samples in Figure 3 and Figure 4. The examined frequency range is defined between 0 and 6000 Hz, so that several bending eigenfrequencies can also be identified for the thicker beam samples. A measuring point grid of 4 × 39 (=156) points is selected for all measurements.

Test measurements show that PLA already has high inherent damping, compared to steel and aluminum. The broadband excitation and measurement of the entire frequency range lead to noise in the higher frequency range and overloads at lower frequencies. For this reason, the frequency range is subdivided into smaller frequency sections of 1000 Hz. Each of these frequency ranges is excited and measured separately. The resulting frequency response of the beam over the entire frequency range is subsequently combined. The individual frequency sections and the signal types used are summarized in Table 3.

### 3.2. Material Parameter Identification for Static Case

**Figure 5:**
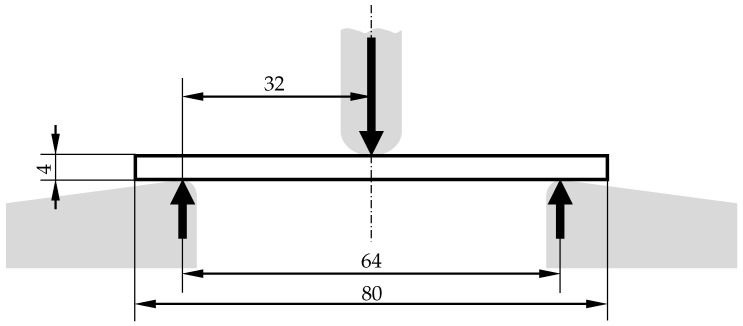
Schematic setup of the three-point bending test with dimensions (in mm).

In order to determine the flexural modulus of the additively manufactured samples test specimens for the bending test are manufactured according to DIN EN ISO 178 by using the process parameters shown in Table 1. The dimensions of the specimens are set to 80 × 10 × 4 mm^3^ and the span is selected to be 64 mm. In Figure 5 the test setup with the beam specimen between the supports and the compression fin is schematically illustrated.

The specimens are tested with a speed of 2 mm/min. The results of the bending test are shown in Table 4. In addition to the values determined for the flexural modulus, the standard deviation is specified. It is to be expected that the flexural modulus will change with increasing frequency. The determined static value of the flexural modulus provides an orientation value between the 3 mm and 6 mm beam at 0 Hz for the fitting of the frequency-dependent modules using numerical simulations.

### 3.3. Results

In this section the experimental results are presented and compared. To compare a quantity independent of the force excitation and equivalent to an energy quantity, the mean squared admittance $h^{2}$ is calculated according to Equation (1).

(1) $$\begin{array}{c} h^{2}\left(f\right)=10\;\log_{10}\left(\sum_{i=1}^{N_{p}}\frac{{\left(\frac{\left|v_{i}\left(f\right)\right|}{\left|F_{i}\left(f\right)\right|}\right)}^{2}}{N_{p}}\right)dB \end{array}$$

In Equation (1), $v_{i}$ are the velocities of each surface point *i* and $F_{i}$ the exciting force. These parameters are frequency-dependent, which also results in a $h^{2}$ value dependent on frequency *f*. $N_{p}$ is the number of surface points.

**Figure 6:**
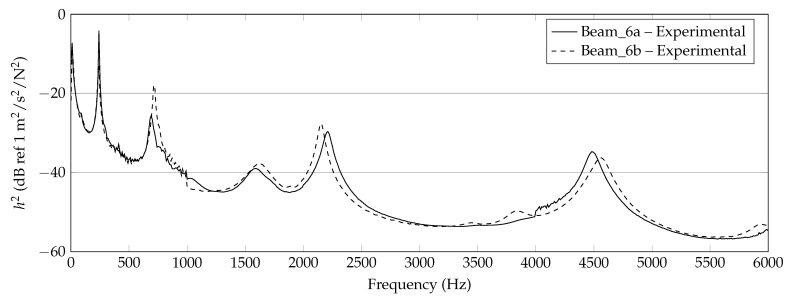
Experimental results of 6 mm beam measurements (Beam_6a, Beam_6b).

**Figure 7:**
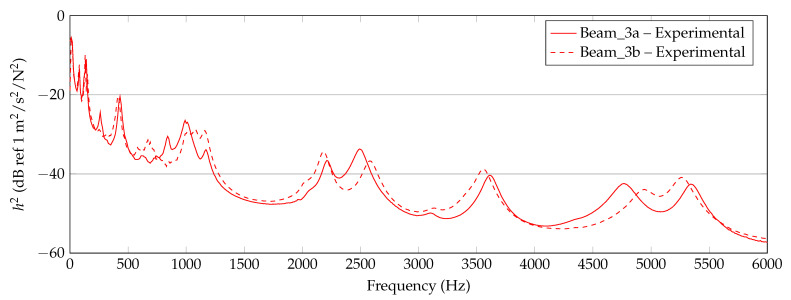
Experimental results of 3 mm beam measurements (Beam_3a, Beam_3b).

**Figure 8:**
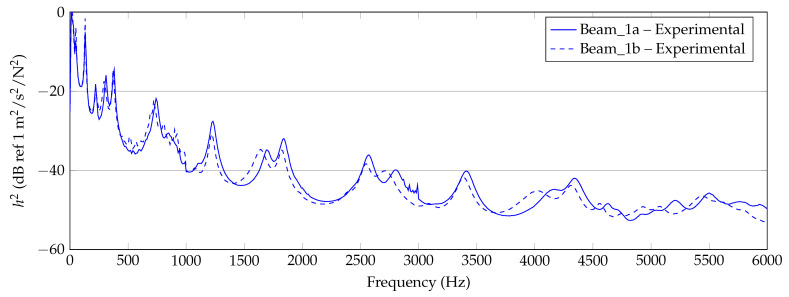
Experimental results of 1 mm beam measurements (Beam_1a, Beam_1b).

In Figure 6, Figure 7 and Figure 8, the experimentally determined frequency responses functions $h^{2}\left(f\right)$ of the different beam types are shown. In the diagrams, a comparison of the frequency response of two beams with similar thickness is shown, respectively. In the lower frequency range up to approximately 500 Hz, deviations between the curves can hardly be detected. In the higher frequencies, some deviations in the position of the peaks and the height are visible. Reasons are assumed in the manufacturing process and in the experimental data collection. Again, this illustrates the sensitivity of the frequency response of additively manufactured structures to process parameters and points out the necessity of a suitable procedure for material parameter identification. However, the differences of the curves seem not as significant as completely different homogenized material parameters are expected.

## 4. Parameter Study by Numerical Investigation

A mechanical model is built and numerically solved in Section 4.1 in order to predict the dynamic behavior of the beams. For each beam, a parameter study is conducted in Section 4.2 to yield the best fitting parameters in the frame of linear elasticity and structural damping for the different thicknesses.

### 4.1. Mechanical Model and Numerical Solution

The major focus of this work is laid on a potential layer-effect within additively manufactured structures with varied thickness *t* as required by typical ABHs. Layer-effect means that elastic material parameters (here: Young’s modulus and loss factor) may change in dependency on the number of printed layers within the manufacturing process. The studies are explicitly limited to the base material of such structures which is PLA in this case. If the PLA material is thicker, different heat inputs are expected which may influence the modeling parameters to be chosen. With that background in mind, the beams are modeled by a 3D continuum with three translational degrees of freedom (dof). This way, the model is extendible to a layer-wise investigation (of the base PLA material only) in future work and a damping measure within the ABH area can be added easily. Linearity is assumed as the measurements show small deflections. Based on experiences, half the mass of the force sensor ($m_{\mathrm{Sensor}}$ = 2 × 0.012 kg) is considered as concentrated mass at the excitation point.

A structured mesh by 27-node hexahedrons with quadratic ansatzfunctions (Lagrangian type) is applied to the 3D continuum and solved in the frequency domain with a frequency step size of Δf = 20 Hz using the institute’s in-house implementation elPaSo [34]. A convergence study is conducted for each beam thickness (1, 3 and 6 mm) in the higher frequency range (4000–6000 Hz). As a criterion, the maximum error of the mean squared admittance $\Delta h^{2}$ (Equation (1)) at all frequency steps $f_{i}$ must be smaller than 0.1 dB. Under this assumption, it is expected that the material parameters are identified exactly enough in the frame of the engineering task.

**Figure 9:**
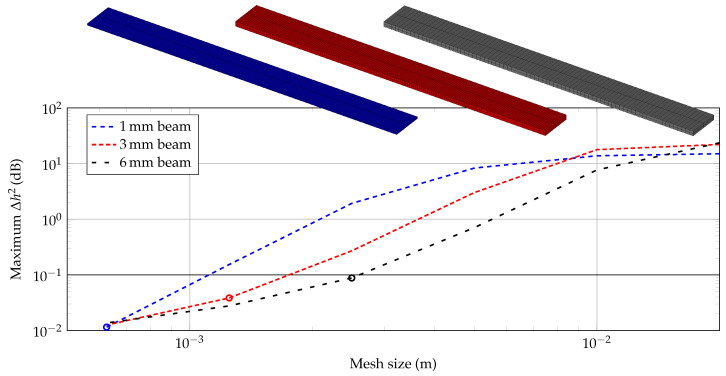
Maximum error in dependency on mesh sizes for each beam specimen with two elements over the beam’s thickness (chosen mesh size marked with circle).

By preliminary studies in [27] and the static bending test in Section 3, an estimation of the minimum flexural modulus (Young’s modulus is called a flexural modulus as bending waves are dominant in the structures applied here.) for PLA is known. These values are round down to *E*_0_ = 3 × 10^9^ N/m^2^ in order to receive a conservative mesh size by the convergence study. Applying $E_{0}$ to the three thickness setups t={1,3,6} mm, the mesh size is reduced systematically, until $\Delta h^{2}$ is smaller than 0.1 dB. In Figure 9, the maximum error is plotted in dependency on the mesh size. The chosen mesh size for each beam is further marked in the figure. Finally, this results in FE models with 12 k, 24 k and 48 k dof for the 6, 3 and 1 mm beam, respectively. The mesh is shown above Figure 9.

**Figure 10:**
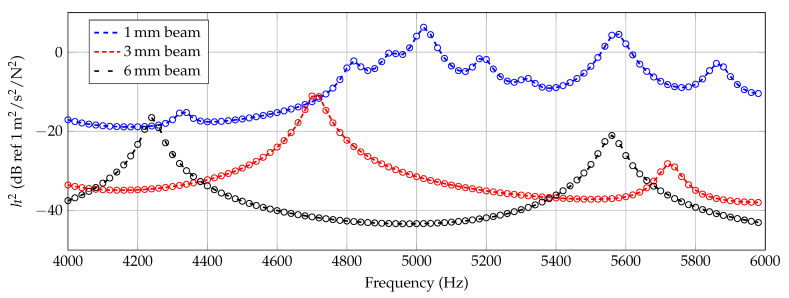
Comparison of $h^{2}$ for two elements (dashed line) and three elements (marks) in thickness direction for each beam.

For the FE meshes applied for the convergence study in Figure 9, two elements (five nodes) over the thickness have been applied. In Figure 10, a comparison with three elements (seven nodes) over the thickness is plotted.

The curves do not show significant differences, hence, the mesh sizes as shown in Figure 9 are applied for the parameter fitting and two elements over the beam’s thickness are considered in the model. The convergence study is a crucial basis for the parameter fitting in Section 4.2 as the authors want to exclude any significant side effects like numerical errors.

In order to speed up the parameter study itself, a reduced-order model (ROM) is derived on the basis of the full order model (FOM). For the model order reduction (MOR) process, a first-order Krylov subspace method based on moment matching is used, since the system is proportionally damped [35]. As mentioned in [35], the obtained ROM is valid for variation of the Rayleigh damping coefficients and therefore also valid for a variation of the flexural modulus, which is just a linear factor for the stiffness matrix. Multi-point moment matching is applied to construct a global basis, which yields a small error over the entire frequency domain [36]. One expansion point is set every 1000 Hz (including 0 Hz and 6000 Hz) while the matched moments are increased until the maximum error $\Delta h^{2}$ is smaller than 0.1 dB. A Gramm–Schmidt orthogonalization is conducted at each step to construct an orthonormal basis and perform a vector-wise deflation strategy. For the convergence tests of the ROM, a frequency step size of Δf = 20 Hz is chosen. The loss factor is set to a conservative value of η = 0.001. In Table 5, the resulting necessary moments are documented—the number of matched moments is kept equal for all seven development points. The parameter fitting is conducted using the ROM while the final results are again computed by the FOM.

### 4.2. Parameter Identification

As mentioned above, the parameter space is discretized and studied entirely. For *E* and η, the ranges are set according to Table 6.

For the identification of optimal parameters, two criteria are applied comparing the experimental curve $h_{\alpha }^{2}$ and the numerical curves of $h_{\beta }^{2}$. The first criterion $\delta_{1}$ is the sum of errors in dB at each frequency sampling points *i* of the total points $N_{f}$ according to Equation (2). The optimal response is assumed to be the one leading to min($\delta_{1}$).

(2) $$\begin{array}{c} \delta_{1}=\sum_{i=1}^{N_{f}}\left|h_{\beta ,i}^{2}-h_{\alpha ,i}^{2}\right| \end{array}$$

As second criterion, Frequency Response Assurance Criterion (FRAC) is applied comparing two frequency responses by scalar multiplication and normalization. A FRAC value of 1 identifies curves with identical course. The absolute level is ignored by FRAC. In Equation (3), the criterion is defined according to [37] and adopted for the application here. The energy quantity $h^{2}$ is directly taken for FRAC with $H_{\alpha /\beta }=10{\;}^{h^{2}/10}$.

(3) $$\begin{array}{c} \mathrm{FRAC}=\frac{{\left(H_{\alpha }{\left(f\right)}^{T}\cdot H_{\beta }\left(f\right)\right)}^{2}}{\left(H_{\alpha }{\left(f\right)}^{T}\cdot H_{\alpha }\left(f\right)\right)\left(H_{\beta }{\left(f\right)}^{T}\cdot H_{\beta }\left(f\right)\right)}. \end{array}$$

#### 4.2.1. Frequency-Independent Parameters

**Figure 11:**
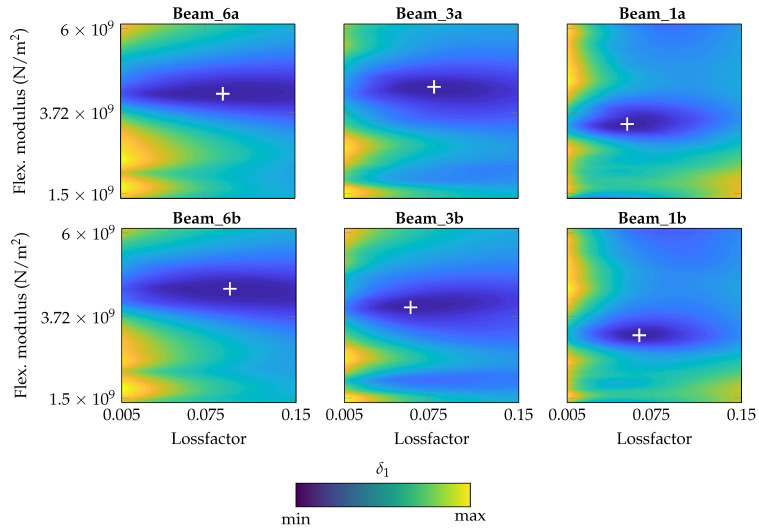
$\delta_{1}$ contour plot for each specimen with identified optima under variation of *E* and η (frequency-independent, constant values).

As the first step, constant parameters *E* and η are applied to the entire frequency range. As criterion, $\delta_{1}$ is applied. In Figure 11, the results are shown for all six specimens separately. For each beam, a best-fitting combination of constant *E* and constant η can be identified which are marked, respectively.

In Table 7, the resulting parameter combinations are listed. A large variation of the resulting constant parameter combinations can be clearly seen which may be a result by the thickness difference or process parameters in general. However, slight tendencies in dependence on the thickness can be observed. With decreasing thickness, the homogenized flexural modulus *E* and the homogenized loss factor η seem to be decreased as well.

#### 4.2.2. Frequency-Dependent Parameters

**Figure 12:**
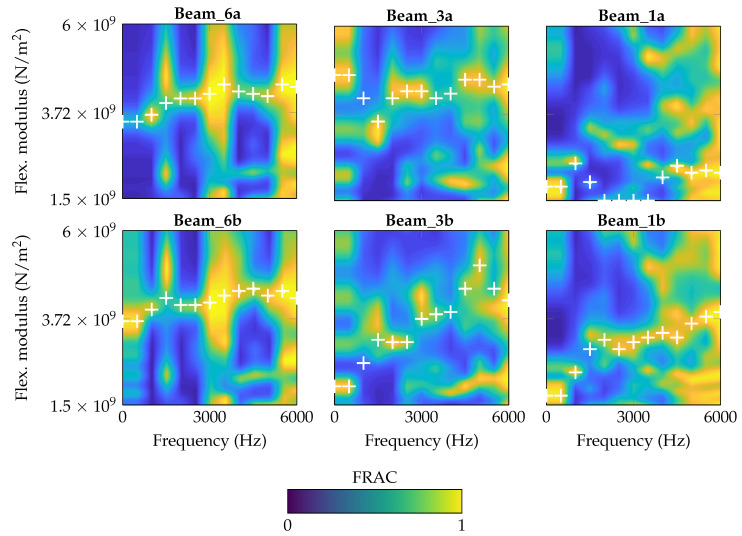
FRAC contour plot for each specimen with identified optima under variation of *E* (frequency-dependent, constant value for η).

The frequency-independent material parameters for each specimen (Table 7) are set as basis for the frequency-dependent material parameter identification for *E*. This means for the following parameter study, a constant η is continuously applied for each beam. Similar to the previous studies, *E* is varied, but now, FRAC is applied as the criterion. Every 500 Hz, a sectionwise FRAC is determined using a range of ±500 Hz. For each sample point (every 500 Hz), the maximum FRAC value is assumed to indicate the best-fitting flexural modulus. Discontinuities in E(f) are suppressed in the identification process by allowing only a maximum difference of 0.6 × 10^9^ N/m^2^ from one sample to the next. The motivation is to avoid non-physical jumps. Finally, the contour plots in Figure 12 are created which show the FRAC distribution over frequency and flexural modulus. The identified values are marked by the +. On the first view, the procedure works quite well with the exception of Beam_1a and Beam_3a. For these specimens, the identified values are not laying on a recognizable curve as FRAC is indicating several best-fitting flexural moduli for one frequency sample.

**Figure 13:**
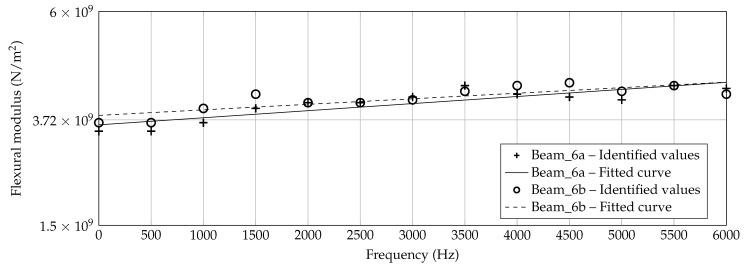
Identified flexural moduli by Figure 12 for Beam_6a/b with fitted linear curve.

**Figure 14:**
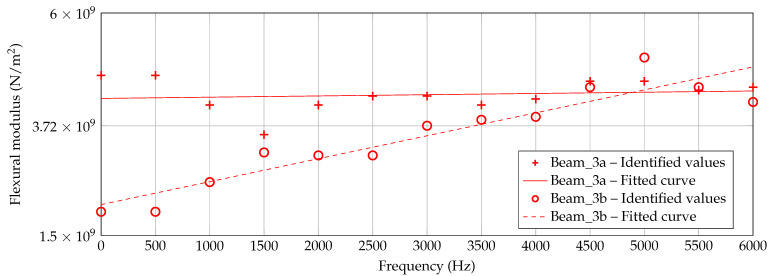
Identified flexural moduli by Figure 12 for Beam_3a/b with fitted linear curve.

**Figure 15:**
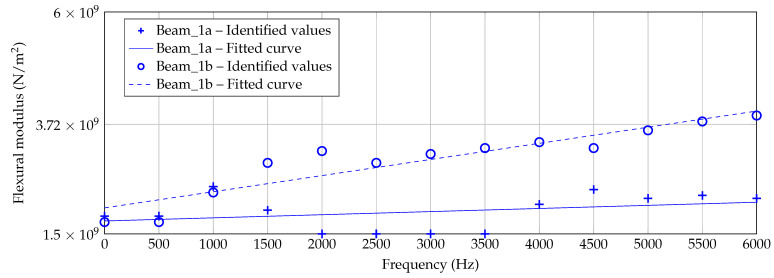
Identified flexural moduli by Figure 12 for Beam_1a/b with fitted linear curve.

However, in Figure 13, Figure 14 and Figure 15, the identified data points are plotted for each beam thickness, respectively. By use of the data points, a linear curve fit is applied in order to receive a practicable mean curve. For Beam_6a and Beam_6b, the two resulting curves are quite similar which indicates a robust manufacturing and identification process. For Beam_3a/b and Beam_1a/b, the two curve are different. According to Figure 12, some outliers and the explanation above may be reasons for this deviation.

The identification process could be improved by a more robust application of the criterion, for instance by a consideration of several dof as intended by [37]. This way, deflection shapes may be matched better and flexural moduli matching different peaks in the $h^{2}$ curve are excluded inherently. Keeping the problems by the identification process in mind, nevertheless an increasing flexural modulus can be observed with increasing frequency for all three thicknesses and all six specimens. Furthermore, the authors exclude a significantly high error due to the measurement setup, as Figure 7 and Figure 8 show a quite well agreement of the two specimen’s $h^{2}$ curves, respectively.

**Figure 16:**
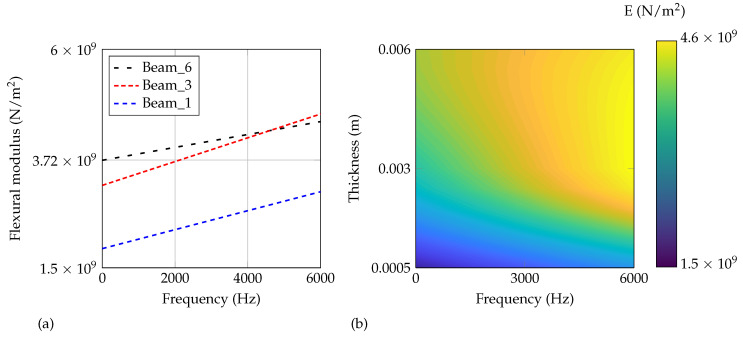
Overall fitted (**a**) linear curves for the three different manufactured thicknesses and (**b**) flexural modulus in dependency on frequency and thickness.

Finally, all sampling points by Figure 12 are used for a fitting per thickness. In Figure 16, the overall fitted linear curves (a) and the distribution of *E* in dependency on frequency and thickness (b) are shown. For the contour plot (b), a piecewise cubic interpolation including extrapolation is applied for the thickness-dimension. Differently, a linear fitting as shown in Figure 16a is applied for the frequency dimension. As motivated in Section 1, a dependency of the flexural modulus on the additively manufactured thickness can be clearly observed. Generally speaking, the flexural modulus decreases with the decreased thickness of the structure. For 0 Hz, the range is between 1.9 × 10^9^ N/m^2^ and 3.7 × 10^9^ N/m^2^ which is about twice the value due to a 6 times thicker beam. The sensitivity reduces comparing the 3 mm beam and the 6 mm beam which indicates a non-linear behavior of the flexural modulus in dependency on the thickness. For ABH structures, besides the usual stiffness reduction by a lowered thickness, an additional effect can be expected by the manufacturing process itself. Considering this effect in mechanical models, a more precise and reliable prediction may be the result.

**Figure 17:**
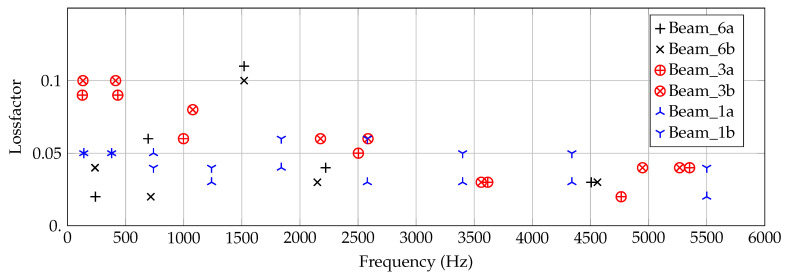
Identified loss factors η for all six specimens in dependency on frequency *f*.

In the second step, the loss factors η are identified by the use of the frequency-dependent flexural moduli. For each significant peak in the experimental curve, the frequency range around (±10%) is investigated with a Δf = 1 Hz. By optical checks and manual adjustment, the loss factors are adapted in a way that the peak levels fit. An automatized routine using $\delta_{1}$ as the criterion is hardly realizable as a slight shift in frequency lead to high differences though the amplitude of close peaks might be similar. This means the results shown in Figure 17 may give only tendencies.

From 2000 Hz, similar loss factors between 0.02 and 0.06 are yielded by all six beams, independent of their thickness. The tendency of all curves shows a slight decrease towards higher frequencies. Below 2000 Hz, a spreading of the values are visible in dependency on thickness. This spreading cannot be identified to be systematic. For example, for both of the 3 mm beams, the homogenized η is increasing up to 0.1 towards lower frequencies.

In opposite, η shows a maximum for both 6 mm beams at around 1500 Hz. This behavior has not been expected and may be assigned to unknown effects based on the micro-structure. Due to the manufacturing process, the layers might act similar to a constraint layer damping. However, at this stage this cannot be further emphasized as only two 6 mm beams are available. In addition, the experimental setup might significantly influence some of the peaks.

### 4.3. Final Results

**Figure 18:**
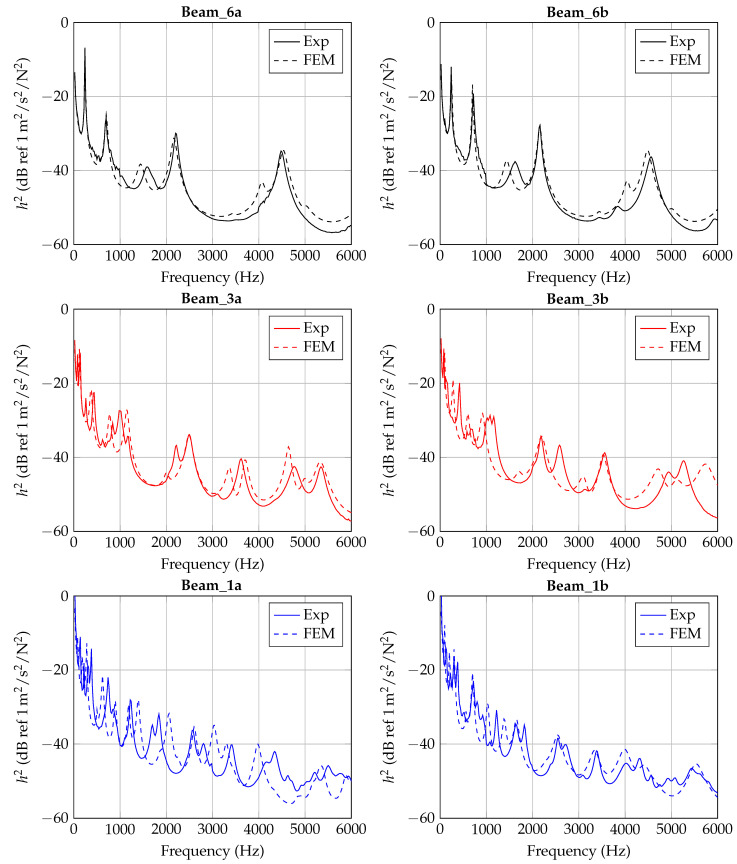
Experimental and numerical response of each specimen with frequency-dependent material parameters *E* and η.

The identified frequency-dependent material parameters by Section 4.2 are applied to each beam specimen and solved with Δf = 20 Hz. For each specimen, the corresponding fitting curve by Figure 13, Figure 14 and Figure 15 are considered. In Figure 18, $h^{2}$ is plotted in comparison with the experiment for each specimen, respectively. The results show a quite good agreement for Beam_6a/b, but with decreasing thickness (Beam_3 and Beam_1) the agreement gets worse. Thinner beams are expected to show higher sensitivities to the manufacturing process and the material parameter identification. Nevertheless, the tendency of a decreased flexural modulus with decreased thickness seems to be appropriate for the problem.

## 5. Conclusions

The aim of the paper is to provide a procedure to identify material parameters of additively manufactured structures for robust and reliable dynamic simulations. AM provides great design freedom but at the same time makes valid mechanical modeling more challenging. As an illustrative example, the procedure is shown in the context of 3D-printed ABHs by using material extrusion. As ABH structures require a continuously adapted thickness profile, the dependency of homogenized material parameters on the thickness of additively manufactured beam structures is studied.

A dependency of Young’s modulus and the loss factor on frequency and thickness can be observed based on the parameter fitting of the 3D model. The homogenized Young’s modulus is decreased with a decreased thickness of the printed structure. Quantitatively, a doubling of the value can be identified due to a change from 1 mm to 3 mm thickness. A change from 3 mm to 6 mm induces a slight change which is no longer systematically. It is assumed that the flexural modulus converges with the thickness as the heat input becomes more homogeneous. The main findings can be summarized as follows:

- dependence of homogenized material parameter (Young’s modulus, loss factor) on frequency and thickness
- Young’s modulus decreases significantly with decreasing thickness

In order to improve the material parameter identification, further studies should focus on an improved criterion comparing the responses and the deflection shapes and an investigation of the inherent uncertainties by measurements and the manufacturing process. For this purpose, a larger number of samples should be analyzed. This way, mechanical models considering uncertain parameters may be applied for a robust design process of additively manufactured structures.

The method presented here is also universally applicable to other additive manufactured materials. Due to the combination with the model order reduction, even more complex fittings can be handled. In this way, it is possible to use the manifold possibilities of AM to optimize the performance of acoustic measures.

## Figures and Tables

**Table 1:** Utilized process parameters for manufacturing of the test specimens.

Material	PLA
Temperature Build Platform	60 °C
Temperature Nozzle	215 °C
Layer Thickness	0.0002 m
Raster Angle	±45°
Perimeter Shells	2
Flow Rate	105%
Infill Percentage	100%
Extrusion Width	0.0004 m
Extrusion Speed	0.05 m/s

**Table 2:** Known geometrical and material data of specimens.

Specimen	Length (m)	Width (m)	Thickness (m)	Mass (kg)	Effective Density (kg/m^3^)
Beam_6a	0.200	0.020	0.0063	0.03081	1222.6
Beam_6b	0.200	0.020	0.0062	0.03067	1236.7
Beam_3a	0.200	0.020	0.0032	0.01613	1260.2
Beam_3b	0.200	0.020	0.0033	0.01643	1244.7
Beam_1a	0.200	0.020	0.0012	0.00588	1225.0
Beam_1b	0.200	0.020	0.0012	0.00579	1206.3

**Table 3:** Overview of frequency boundaries and signal type for separately excited and investigated frequency ranges.

Frequency Range (Hz)	Signal Type
0–1000	Pseudo Random
1000–2000	Sweep
2000–3000	Sweep
3000–4000	Sweep
4000–6000	Sweep

**Table 4:** Flexural modulus (static) of PLA according to DIN EN ISO 178.

Material	PLA
Flexural modulus	3196.75 × 10^6^ N/m^2^
Standard deviation	68.98 × 10^6^ N/m^2^

**Table 5:** Number of matched moments in the reduced-order model (ROM) in order to ensure a maximum $\Delta h^{2}$ < 0.1 dB for the parameter identification.

Model	Moments Matched
6 mm beam	2
3 mm beam	3
1 mm beam	5

**Table 6:** Parameter space including assumed constants.

Parameter		Unit	Range	Delta
Flexural modulus	*E*	N/m^2^	1.5 × 10^9^–6.0 × 10^9^	0.06 × 10^9^
Loss factor	η		0.005–0.15	0.005
Density	ρ	kg/m^3^	constant (see Table 2)	
Poisson’s ratio	ν		constant (0.35)	

**Table 7:** Frequency-independent identified parameter combinations with minimum $\delta_{1}$.

Specimen	Thickness (m)	Flexural Modulus (N/m^2^)	Loss Factor
Beam_6a	0.0063	4.20 × 10^9^	0.090
Beam_6b	0.0062	4.44 × 10^9^	0.095
Beam_3a	0.0032	4.38 × 10^9^	0.080
Beam_3b	0.0033	3.96 × 10^9^	0.060
Beam_1a	0.0012	3.42 × 10^9^	0.055
Beam_1b	0.0012	3.24 × 10^9^	0.065

## Data Availability

Data sharing is not applicable to this article.

## Abbreviations

The following abbreviations are used in this manuscript:

3D	Three-dimensional
ABH	Acoustic Black Hole
AM	Additive manufacturing
$\delta_{1}$	First error criterion
dof	degrees of freedom
*E*	Young’s modulus
elPaSo	Elementary parallel solver (in-house code)
η	Loss factor
*f*	Frequency
*F*	Exciting Force
FOM	Full order model
FRAC	Frequency Response Assurance Criterion
$h^{2}$	Mean squared admittance
*H*	Frequency response function
MEX	Material extrusion
MOR	Model order reduction
MSA	Mean squared admittance
$N_{f}$	Number of frequency points
$N_{p}$	Number of surface points
ν	Poisson’s ratio
PLA	Polyactic acid
ϱ	Density
ROM	Reduced order model
*t*	Thickness of beam specimens
TPU	Thermoplastic polyurethane
*v*	Surface velocity
